# Are MRI-defined fat infiltrations in the multifidus muscles associated with low back pain?

**DOI:** 10.1186/1741-7015-5-2

**Published:** 2007-01-25

**Authors:** Per Kjaer, Tom Bendix, Joan Solgaard Sorensen, Lars Korsholm, Charlotte Leboeuf-Yde

**Affiliations:** 1 The Back Research Center, Backcenter Funen, Part of Clinical Locomotion Science, University of Southern Denmark, Denmark; 2 Department of biostatistics, University of Southern Denmark, Denmark

## Abstract

**Background:**

Because training of the lumbar muscles is a commonly recommended intervention in low back pain (LBP), it is important to clarify whether lumbar muscle atrophy is related to LBP. Fat infiltration seems to be a late stage of muscular degeneration, and can be measured in a non-invasive manner using magnetic resonance imaging. The purpose of this study was to investigate if fat infiltration in the lumbar multifidus muscles (LMM) is associated with LBP in adults and adolescents.

**Methods:**

In total, 412 adults (40-year-olds) and 442 adolescents (13-year-olds) from the general Danish population participated in this cross-sectional cohort study. People with LBP were identified through questionnaires. Using MRI, fat infiltration of the LMM was visually graded as none, slight or severe. Odds ratios were calculated for both age groups, taking into account sex, body composition and leisure time physical activity for both groups, and physical workload (in adults only) or daily bicycling (in adolescents only).

**Results:**

Fat infiltration was noted in 81% of the adults but only 14% of the adolescents. In the adults, severe fat infiltration was strongly associated with ever having had LBP (OR 9.2; 95% CI 2.0–43.2), and with having LBP in the past year (OR 4.1; 1.5–11.2), but there was no such association in adolescents. None of the investigated moderating factors had an obvious effect on the OR in the adults.

**Conclusion:**

Fat infiltration in the LMM is strongly associated with LBP in adults only. However, it will be necessary to quantify these measurements objectively and to investigate the direction of this link longitudinally in order to determine if the abnormal muscle is the cause of LBP or *vice versa*.

## Background

It is generally believed that muscular insufficiency and low back pain (LBP) are linked, even though the main direction of this link is unclear. Does insufficient muscular strength or control cause LBP or does LBP affect the muscles and their function?

There are other uncertainties. Although several authors have suggested that in some people, specific training involving specific muscles [1-5] can reduce the symptoms [6-8] and prevent recurrence,[9] the mechanism through which these effects are mediated is unknown. It would therefore be valuable to discover whether there really is a link between certain muscles and LBP, and to determine the nature of this link.

Histological studies have shown a change in distribution of fiber types and a reduction of muscle size in patients with chronic LBP[10,11] and intervertebral disc herniation. [12-14] In several imaging studies, back pain has been investigated in relation to muscle size, [15-26] perfusion,[27] and fat infiltration.[15-19,21,22,25,28-32] Although the results point towards an association between LBP and morphological muscle changes, further studies using larger and unbiased study samples with a clear definition of the muscular observations are necessary.

Based on our understanding of the scientific literature in this area, we hypothesised that LBP leads to altered neuromuscular functioning, which in turn results in changes in muscle histology, seen as atrophy. However, the cross-sectional area of the muscle may not decrease, due to fatty infiltration in the muscle bundle. Fat infiltration can be seen on magnetic resonance imaging (MRI), and in human cadaver studies, MRI has been shown to be a valid method of identifying muscle volume[33] and the amount of fat in human skeletal muscle.[34,35]

Therefore, the aims of this study were: (i) to determine the prevalence of fat in the LMM at the three lower lumbar levels in 13-year-old and 40-year-old participants; and (ii) to investigate whether there is an association between such fat infiltration and LBP, taking into account the possible effects of sex, body mass and leisure time activity in both groups, and physical workload (adults only) or bicycling (adolescents only).

## Methods

### Participants

MRI scans of approximately 850 13-year-old and 40-year-old Danish participants were collected in a population-based study. Information on their LBP status was obtained, making it possible to study the association between LBP and the morphology of muscles in two large distinct age groups. In addition, there was information on a number of lifestyle factors, which made it possible to relate this link to the potentially modifying/confounding effect of sex, obesity, type of work, and physical activity.

We studied population-based cohorts of adults and adolescents living in the county of Funen, Denmark. This county has approximately 500 000 inhabitants (about 10% of the total Danish population). The adults were randomly selected (every ninth person of 40 years of age born in Denmark was chosen) by the Central Office of Civil Registration to be representative of this age group. The adolescents were living in the municipality of Odense (a rural/city area of 184 000 inhabitants in county of Funen, Denmark). They had previously participated in the European Youth Heart Study, and were selected by a cluster sampling method, taking into account socioeconomic factors and rural/urban distribution.[36] At the time of sampling, there were approximately 6500 40-year-olds and 5300 13-year-olds living in the county of Funen. We expected the samples to be representative of their respective age groups in the county of Funen and Denmark. In total, 412 40-year olds (199 men and 213 women) and 442 13-year-olds (206 boys and 236 girls) participated in the study. This study was part of a larger investigation of lumbar MRI variables. Details of the sampling and study procedures and further details on the representativeness have been published elsewhere. [37-39] Permission for the study was granted by the local ethics committee and for the database by the Danish Data Protection Agency. All participants (as well as the parents of the adolescents) gave their informed consent.

### MRI methods

**Figure 1:**
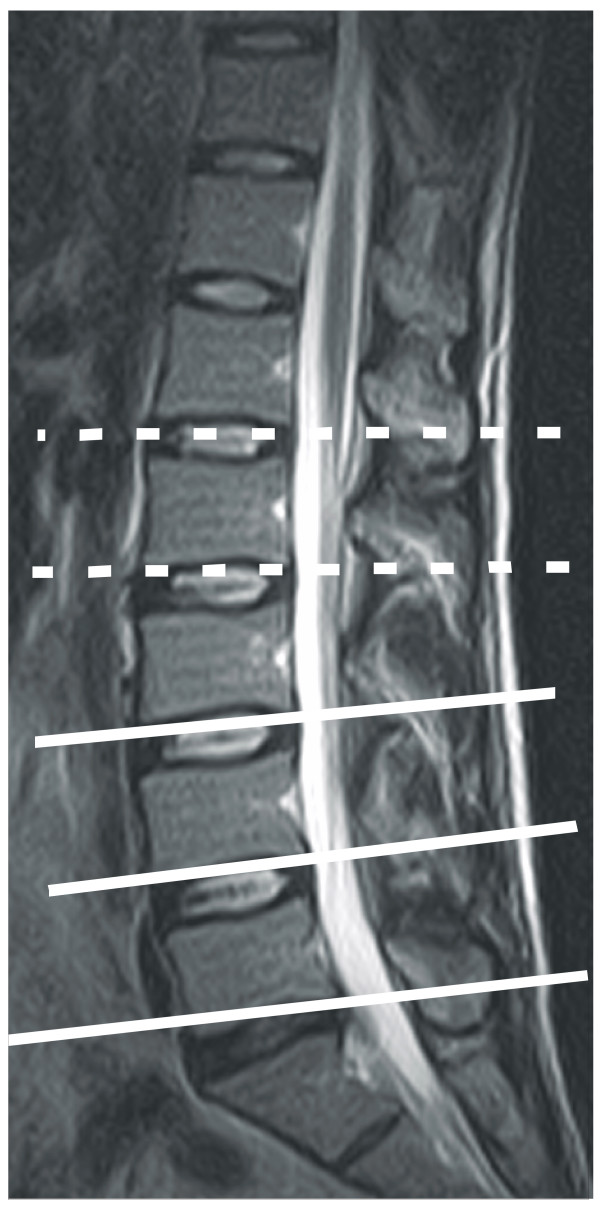
The positioning of the axial images. Only the three lower levels were included in the analyses.

In a pilot study, we investigated several methods and MRI protocols in order to achieve optimal distinction between the different muscles in the lumbar spine.[29,40] An axial T1-weighted spin echo (300/26 repetition time/echo time, 120 × 256 matrix, 280-mm field of view, and 4-mm section thickness) was decided for this part of the study. The MRI system was an open low-field 0.2 T MR unit (Magnetom Open Viva, Siemens AG, Erlangen, Germany), and a body spine surface coil was used. The lumbar spine was imaged at the five lumbar levels. Slices were positioned from T1 (and in adolescents T2) median sagittal images tangentially to the posterior caudal corner of the upper vertebral body and perpendicularly to the surface of the lumbar muscles as illustrated in Figure 1. For the purposes of this study, only the three lower segments were evaluated.

Although several authors have used MRI to evaluate the erector spinae group,[26,31,41] only one study focused specifically on the LMM.[29] Our method varies from that study in terms of the positioning of images.

### Visual evaluation

**Figure 2:**
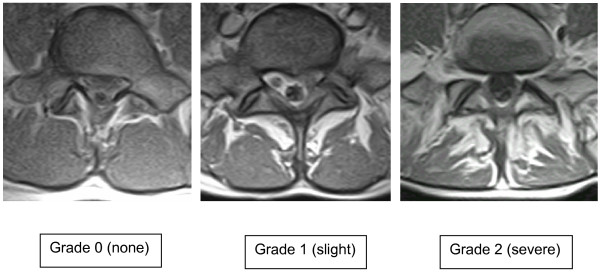
Examples of amounts of fat in the lumbar multifidus muscles as seen on axial T1- weighted magnetic resonance imaging scans. These were rated as grade 0 if normal condition; grade 1 for slight fat infiltration (10–50%), and grade 2 for severe fat infiltration (>50%).

Fat infiltration of the LMM was visually graded using the standard criteria: "normal" for estimates of 0–10% fat within the muscle, "slight" for 10–50% fat, and"severe" for >50% fat (Figure 2). The grading system was adapted from previous studies using low-field MRI [25,29] and tested for intra-observer and interobserver agreement in two reliability studies:[37,42] A sample of 50 sets of images from the adult cohort was read by two consultant radiologists, and another 50 sets of images from the adolescent cohort was read by a consultant radiologist and a doctor with extensive experience in MRI. Intra-observer agreement was very high (κ = 0.86; 95% CI 0.74–0.99), and inter-observer agreement substantial (κ = 0.58; 0.49–0.67) when visually evaluating fat infiltration in the LMM in adults.[42] For the adolescents, intra-observer agreement was κ = 0.28 (0.19–0.39), but there were too few positive findings to calculate kappa values for the interobserver agreement.[37] However, the percentage interobserver agreement was 92%. All evaluations of LMM used in this study were performed by a single radiologist (JSS), who also took part in the previous interobserver and intra-observer reproducibility studies. The assessor was blinded to all information about the study participants obtained from questionnaires and clinical examination.

### Defining LBP

LBP was defined in the adults from questionnaires that have previously been used in Danish populations. [43-45] For the adolescents, additional information from an interview was used.[36] In the adults, answering "yes" to one of the questions: "Have you ever had low back trouble?" or "Have you ever had pain radiating into one or both legs?" defined "LBP ever". Reporting at least 1 day with LBP in the previous year was defined as "LBP year". In the adolescents, answering "yes" to the questions: "Have you had back pain within last year?" while pointing to the lower back defined "LBP year", and "Have you had back pain within the past week/month?" while pointing to the lower back defined "LBP week/month", as appropriate. The validity and recall bias for these types of instruments have previously been addressed.[36,45,46] In the present study, information from interview and questionnaires regarding different definitions of LBP in children was compared by cross tabulation and calculation of percentage agreement and kappa values. The most recent LBP ("LBP week") had the highest percentage agreement (92%; κ = 0.53) and the "LBP month" variable was found to be most reliable (86%; κ = 0.58).[37]

### Possible effect moderators

Several variables that may influence the presence of fat in the multifidus were considered. In both adults and adolescents, body mass index (BMI) was calculated as the weight in kilograms divided by the square of the height in meters. In the adults, self-reported height and weight were used to calculate BMI. The adolescents had their height and weight measured on the day of the examination. On the basis of the BMI, each person was classified as being of normal body weight, overweight or obese following international criteria for cut-off points.[47] In adults, BMI <25 was normal, 25–29.9 overweight, and ≥30 obese. The corresponding figures for adolescents were <22.5, 22.5–27.5 and ≥27.5, respectively (cut-off points were set as the mean of the internationally suggested cut-off points for boys and girls at the age of 13 years).[47]

**Figure 3:**
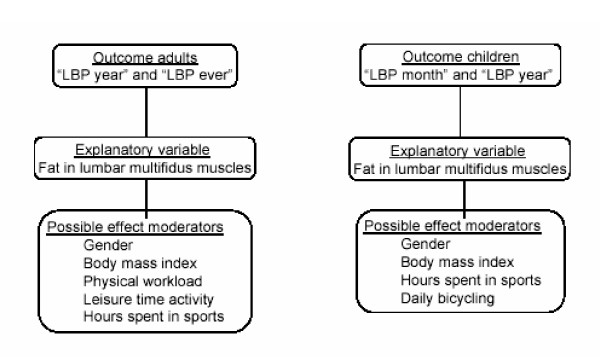
An overview of the study design and the variables.

In both groups, information in the questionnaires about weekly hours of exercise was grouped into four categories: 0 (<2 hours of exercise per week), 1 (2–4 hours), 2 (4–6 hours), and 3 (>6 hours).[48] For adults, data on physical workload were classified into four categories based on self-reporting by choosing one of the categories in the questionnaire: 1 (sedentary), 2 (sedentary/walking), 3 (light physical), and 4 (heavy physical).[49] Reports of leisure-time activities were classified in the same way into four categories: 1 (not active), 2 (walking/biking >4 hours per week), 3 (active in sports >3 hours per week), and 4 (competitive sports). Similarly, for adolescents, hours of daily cycling were also recorded but only daily cycling (yes/no) was used. An overview of the study design and the variables is shown in Figure 3.

### Statistical analysis

The statistical analyses were performed using Stata 8 statistical software (version 8.2); StataCorp LP, College Station, TX, USA.

The prevalence of positive results for fat infiltration in the LMM was listed for each side and level. Data were condensed into a new variable and assigned the values 0 (no fat), 1 (slight infiltration), and 2 (severe infiltration) if present at one or more lumbar levels. To detect effect modification and possible confounding,[50] sex, body weight, and the covariates mentioned above (Figure 3) were tested for: (i) correlations with each other using Spearman correlation analysis, (ii) association with the explanatory variable (fat infiltration) using logistic regression, and (iii) association with the LBP variables. This was done separately for adults and adolescents. The final multivariate models included sex, body weight and the potential moderators/confounders.

The link between fat in the LMM and LBP was investigated using logistic regression. Firstly, logistic regression was performed without considering the grading (grades 1 or 2) of fat infiltration. Secondly, the strength of association for the mild and severe fat infiltration in the LMM was compared with no fat infiltration. The ORs for the more severe findings relative to the milder observations were established using the LINCOM command in Stata, in which the risk difference and CIs were calculated. The data are presented as crude and adjusted ORs with 95% CI. Cross-tabulations of variables were inspected, and significant associations reported.

### Post hoc analyses

Significant differences were observed in prevalence of fat infiltration in the LMM between the sexes in adults. Therefore, all analyses were repeated for men and women separately, i.e. estimates of associations were reported for the effect moderators in relation to fat in he LMM and for the LBP variables. Sex-stratified multivariate analyses in relation to the LBP variables were also reported. No statistically significant associations between fat in the LMM and LBP were found in adolescents.

## Results

### Descriptive data

Complete datasets were available for 409 adults (66% of the invited study sample) and 439 adolescents (80% of the invited study sample). A detailed description of all variables included in the analyses is shown in Table 1, summarised below.

"LBP ever" and "LBP year" were reported by 85% and 70% of the adults, respectively. In adolescents, "LBP year" was reported by 41% and "LBP month" by 22%.

Fat infiltration of the LMM was far more common in the adults; slight fat infiltration was observed in 71% and severe infiltration in 10%, whereas in adolescents these figures were 14% and 0%, respectively (Table 1). Infiltration was most commonly found at the lowest lumbar level, with no difference between left and right, and this did not change with age (data not shown). Female participants had markedly higher prevalence rates of fat in the LMM [90% in women versus 71% in men (p < 0.0001) and 20% in girls versus 6% in boys (p < 0.0001)]. Other prevalence rates for men and women are shown in Table 1.

In adults, 43% were overweight or obese compared with only 16% of adolescents. A fairly regular spread of data was noticed in the four groups of activity at work and in the groups ofhours spent in sports. This was also the case for the adolescents' participation in sports. Regarding physical activity in the adults, few participated in competitive sports and relatively few reported not being active in leisure time. A large proportion of the children cycled daily (85%).

### Correlations and associations

#### Correlations between effect moderators

Several statistically significant correlations between the effect moderators were noted in the adults. However, these were weak (Spearman correlation coefficients (*R*_*s*_) = -0.17 to 0.29). In adolescents, however, there were only two statistically significant associations. "Hours of sports activity" was negatively associated with body weight (*R*_*s *_= 0.14) and sex (girls being one category less active, Wilcoxon test p = 0.0015).

#### Associations between the effect moderators and fat in the LMM

Women were more likely than men to have fat infiltration of any type (OR = 3.7; 95% CI 2.1–6.3). Those adults who reported being active in sports or having heavy physical work, had statistically significantly less severe fat infiltrations (prevalence of 4% and 5% respectively versus the 10% overall prevalence). In adolescents, only sex was positively associated with fat in the LMM (OR = 3.7; 1.9–7.1).

#### Associations between the effect moderators and LBP

As shown in Table 2, none of the effect moderators was associated with LBP in adults. In adolescents, sex was associated with the two LBP variables (girls having the highest prevalence rates of LBP).

#### Associations between fat infiltrations and LBP

As shown in Table 3, there were positive associations between fat infiltration in the LMM and LBP for adults, and the estimates were consistently higher for "LBP ever". The association was strongest (OR = 7.2) for severe vs. no fat infiltration in "LBP ever" but still considerable for "LBP year" (OR = 3.6). These associations slightly increased after controlling for the possible effect moderators (adjusted ORs 9.2 and 4.1, respectively).

In adolescents, no such link was detected. The OR for "LBP year" between slight fat infiltration vs. no infiltration was 1.5 (95% CI 0.8–2.9) and for "LBP month" was 1.5 (0.9–2.6). Final analyses controlling for the effect moderators in bivariate and multivariate analyses did not produce any statistically significant associations.

### Post hoc analyses

#### Associations between effect moderators and fat in the LMM

There were no statistically significant differences in the estimates from the sex-stratified analyses in adults between the effect moderators and fat in the LMM.

#### Associations between effect moderators and LBP

In male participants, no marked deviations were noted from the estimates obtained in the analyses of both male and female participants together. In female participants, however, body weight and weekly hours of exercise became statistically significant and more strongly associated with "LBP ever", and weekly hours of exercise with "LBP year". A further look at the association between hours of sports and LBP among women showed a lower frequency of LBP among those being active for 2–4 hours compared with those who were more active and those who were less active.

#### Sex-stratified associations between fat in the LMM and LBP

As shown in Table 3, the main effect of fat infiltration in the LMM in relation to LBP was found in women. Particularly, the association between severe fat infiltration and LBP variables were stronger in female and weaker in male participants. In female participants, overweight (OR = 2.2 in relation to "LBP year") and time spent in sports (ORs = 1.5 for "LBP year" and 1.8 for "LBP ever") remained statistically significant in the multivariate analyses. In male participants, the estimates of association from multivariate analyses changed slightly compared with those from the combined analyses. All associations with "LBP year" became statistically insignificant. Association with "LBP ever" increased for slight fat infiltration in the LMM, whereas the association for severe fat infiltration in the LMM was reduced and lost statistical significance.

## Discussion

The results of this study provide the first convincing evidence from a large population sample that fat infiltration in the LMM is strongly associated with LBP in adults. This association was not affected by body mass index, type of work, or the level of physical activity during leisure time. However, the associations seem to be more pronounced in women. It will be necessary to investigate in prospective studies whether there is also a causal link, and if so, whether the fat infiltration is the cause of LBP or *vice versa*. Furthermore, it would be useful to confirm whether fat infiltration in the LMM is reversible, as indicated in two previous studies[24,31], and if so, whether this reversibility coincides with improvement of symptoms.

Fat infiltration was identified in 81% of the adult study sample but in only 14% of the adolescents. High prevalence rates have also been reported in previous studies including patients[29] and controls[25], and fat infiltration has previously been noted to be more common in older people.[40] The fact that fat infiltration is more common in adults does suggest that it is the LBP that causes muscle degeneration and that in adolescents, the LBP has not yet lasted sufficiently long to produce such changes. Future longitudinal studies are needed to clarify the extent to which age and LBP contribute to the development of fat in the LMM.

The marked differences in fat in the LMM in males and females may be a result of the well-documented differences in body composition. It appears that the higher proportion of body fat in females is also reflected in the proportion of fat in the LMM. This raises the question as to whether grading should be different for males and females. Furthermore, the high prevalence rate of slight fat infiltration in the LMM indicates a need to change the cut-off point used for grading.

Not only was fat infiltration uncommon in adolescents but there was also no significant association between it and LBP. These results should be interpreted with caution: the intra-observer and interobserver reliability for the adolescents' MRI scans were unsatisfactory, and the validity of the LBP variables debatable. Reasons for the poor reliability in reading MRI may be the long time span between the readings in the intra-observer section and poor consensus in the interobserver section. The observed discrepancies in reporting LBP from interviews and questionnaires probably originates from a lack of understanding of the anatomic area of interest, and from difficulties in defining LBP in each individual. Future studies should carefully address these methodological issues.

A second problem is that it may be difficult to establish the extent of fat infiltration in muscles by mere visual inspection. In view of the encouraging results in this study, the association between fat infiltration in the LMM and LBP should be investigated using an objective quantification method in order to define clinical relevant cut-off points in relation to LBP. This future work should target applicable and clinically relevant redefinitions for visual evaluation that take into account age and sex differences.

Another point of concern is the measure of overweight. BMI was shown not to influence the presence of fat in the LMM. We are inclined to accept this finding, because fat infiltration was found mainly at the L5 level, to a lesser extent at the L4 level, and was virtually absent at the L3 level. If body fat in the obese naturally deposits itself in human muscles, one would expect it to do so evenly throughout the back musculature and not to settle so markedly at the two lumbar levels where most spinal abnormalities generally tend to cluster.[39,51] The fact that fat infiltration is found mainly at these two "trouble areas" tends to indicate that it is the LBP that initiates the muscle changes and not *vice versa*. Nevertheless, it would be prudent to include other descriptions of body fat, such as skin-fold measurements, in future studies, because BMI cannot differentiate between fat contents and muscle:bone ratio.

## Conclusion

Fat infiltration in the LMM is common in adults and strongly associated with LBP, especially in women. It seems to be independent of the body fat estimated using BMI, and to develop in the areas where most degenerative changes are found. In adolescents, fat infiltration is uncommon (particularly in boys) and no significant association with LBP was detected. However, the subjective aspect of this study, in which the amount of fat infiltration was visually determined, makes it necessary to verify the results using an objective method to measure the extent of fat infiltration. In addition, the temporal relationship of fat infiltration needs to be addressed.

## Competing interests

The author(s) declare that they have no competing interests.

## Authors' contributions

PK took part in all elements of the study and drafted the manuscript. TB contributed to the design of study, data interpretation and revision of the manuscript. JSS participated in developing the MRI protocol, the intra-observer and interobserver reliability studies, examined all the MRI images, and reviewed the manuscript. LKK contributed to the study design, participated in the data interpretation and supervised the statistical analyses. CL-Y contributed to the design of the study, data interpretation, and drafting and revision of the manuscript. All authors read and approved the final manuscript.

## Pre-publication history

The pre-publication history for this paper can be accessed here:



## Figures and Tables

**Table 1:** Characteristics for participants (adults and adolescents)

	Adults				Adolescents	
	
			Men	Women		
					
Variables	*n*	(%)	(%)	(%)	*n*	(%)
Sex						
Male	199	(48)	--	--	205	(47)
Female	213	(52)	--	--	234	(53)
LBP variables						
LBP month	--	--			98	(22)
LBP year	289	(70)	(70)	(70)	182	(41)
LBP ever	352	(85)	(85)	(85)	--	--
Fat infiltration in LMM in at least one level						
Grade 0 (none)	78	(19)	(29)	(10)	379	(86)
Grade 1 (slight)	293	(71)	(63)	(79)	60	(14)
Grade 2 (severe)	41	(10)	(9)	(11)	0	(0)
Body weight						
Normal	234	(57)	(46)	(68)	369	(84)
Overweight	131	(32)	(47)	(18)	56	(13)
Obese	44	(11)	(8)	(14)	11	(3)
Workload						
Sedentary	89	(22)	(20)	(24)	--	--
Sedentary/walking	144	(35)	(30)	(40)	--	--
Light physical	92	(22)	(19)	(26)	--	--
Heavy physical	84	(21)	(31)	(11)	--	--
Physical activity						
Not active	59	(14)	(12)	(17)	--	--
Walking/cycling >4 hours	204	(50)	(48)	(51)	--	--
Active sports >3 hours	140	(34)	(38)	(31)	--	--
Competitive sports	9	(2)	(3)	(2)	--	--
Weekly hours of sports activity						
<2	91	(22)	(27)	(18)	117	(27)
2 – 4	107	(26)	(23)	(29)	120	(27)
4 – 6	103	(25)	(24)	(26)	97	(22)
>6	111	(27)	(27)	(27)	92	(19)
Daily bicycling						
Not every day	--	--	--	--	66	(15)
Every day	--	--	--	--	370	(85)

LBP, lower back pain; LMM, lumbar multifidus muscles.

**Table 2:** Associations between the effect moderators and (i) fat in lumbar multifidus muscles and (ii) LBP variables expressed as odds ratios with 95% confidence intervals (OR (95% CI)).

	Adults			Adolescents		
	
Variables	Fat LMM	LBP year	LBP ever	Fat LMM	LBP month	LBP year
Sex	3.7 (2.1–6.3)	0.9 (0.6–1.5)	1.1 (0.6–1.7)	3.7 (1.9–7.1)	1.5 (1.0–2.4)	1.6 (1.1–2.4)
Body weight	0.9 (0.6–1.2)	1.2 (0.9–1.6)	1.3 (0.9–2.0)	0.6 (0.3–1.4)	1.5 (1.0–2.4)	1.4 (0.9–2.2)
Workload	0.9 (0.7–1.1)	1.1 (0.9–1.4)	1.2 (0.9–1.6)	--	--	--
Physical activity	0.8 (0.6–1.2)	0.9 (0.7–1.2)	1.0 (0.7–1.5)	--	--	--
Weekly hours of sports activity	1.0 (0.8–1.3)	1.1 (0.9–1.3)	1.2 (0.9–1.5)	0.9 (0.8–1.1)	1.0 (0.9–1.1)	1.0 (1.0–1.1)
Daily bicycling	--	--	--	0.6 (0.3–1.3)	1.1 (0.6–2.1)	1.5 (0.9–2.6)

Data are expressed as odds ratios (95% confidence intervals).

The estimates denote the OR for each step in those categorical variables that take >2 values.

Fat LMM, fat in lumbar multifidus muscles; LBP, lower back pain; LBP ever, had ever experienced lower back pain; LBP month, had experienced back pain in previous month; LBP year, had experienced back pain in previous year.

**Table 3:** Associations between the amount of fat in the lumbar multifidus muscles and LBP in 40 year-old men (*n *= 199) and women (*n *= 213).

	Unadjusted		Adjusted*	
	
Fat infiltration	LBP year	LBP ever	LBP year	LBP ever
Men and women				
Any	1.7 (1.1–2.6)	2.6 (1.5–4.4)	1.8 (1.2–2.8)	2.9 (1.5–5.5)
Slight (grades 1 vs. 0)	1.5 (0.9–2.5)	2.5 (1.4–4.7)	1.6 (0.9–2.8)	2.9 (1.5–5.5)
Severe (grades 2 vs. 0)	3.6 (1.4–9.7)	7.2 (1.6–32.4)	4.1 (1.5–11.2)	9.2 (2.0–43.2)
Severe (grades 2 vs. 1)	2.5 (1.0–6.1)	2.8 (0.7–12.2)	2.5 (1.0–6.4)	3.2 (0.7–14.1)
Men				
Any	1.4 (0.9–2.5)	2.8 (1.4–5.9)	1.5 (0.8–2.6)	3.0 (1.4–6.4)
Slight (grades 1 vs. 0)	1.6 (0.8–3.0)	3.1 (1.4–6.4)	1.7 (0.9–3.3)	3.4 (1.5–7.7)
Severe (grades 2 vs. 0)	1.9 (0.5–6.6)	5.7 (0.7–46.9)	1.7 (0.5–6.2)	5.7 (0.7–48.0)
Severe (grades 2 vs. 1)	1.2 (0.4–4.0)	1.9 (0.2–15.2)	1.0 (0.3–3.5)	1.7 (0.2–14.2)
Women				
Any	2.4 (1.3–4.5)	2.7 (1.2–6.1)	2.8 (1.4–5.7)	3.2 (1.3–7.9)
Slight (grades 1 vs. 0)	1.6 (0.6–4.1)	2.4 (0.8–6.8)	1.7 (0.7–4.5)	2.7 (0.9–8.1)
Severe (grades 2 vs. 0)	8.3 (1.5–44.5)	9.2 (1.0–84.3)	11.6 (2.0– 66.7)* *	15.1 (1.5–154.3)* *
Severe (grades 2 vs. 1)	5.1 (1.1–22.4)	3.8 (0.5–29.7)	6.7 (1.4–30.8)	5.7 (0.7–46.9)

Data are expressed as odds ratios (95% confidence intervals).

*Adjusted for BMI, physical activity, physical workload, andhours participating in sports;

** time in sports, and body weight remained significant in multivariate analyses.

LBP, lower back pain; LBP ever, had ever experienced lower back pain; LBP year, had experienced lower back pain in previous year.
